# Noise Enhances Action Potential Generation in Mouse Sensory Neurons via Stochastic Resonance

**DOI:** 10.1371/journal.pone.0160950

**Published:** 2016-08-15

**Authors:** Irene Onorato, Giuseppina D'Alessandro, Maria Amalia Di Castro, Massimiliano Renzi, Gabriella Dobrowolny, Antonio Musarò, Marco Salvetti, Cristina Limatola, Andrea Crisanti, Francesca Grassi

**Affiliations:** 1 Institute Pasteur-Cenci Bolognetti Foundation, Dept. Physiology and Pharmacology, Sapienza University, Rome, Italy; 2 Institute Pasteur-Cenci Bolognetti Foundation, DAHFMO-Unit of Histology and Medical Embryology, IIM, Sapienza University of Rome, Rome, Italy; 3 Center for Life Nano Science@Sapienza, Istituto Italiano di Tecnologia, Rome, Italy; 4 Dept. of Neurosciences, Mental Health and Sensory Organs, Sapienza University, Rome, Italy; 5 NeuroMed, Pozzilli, (IS), Italy; 6 Dept of Physics, Sapienza University, Rome, Italy; University of British Columbia, CANADA

## Abstract

Noise can enhance perception of tactile and proprioceptive stimuli by stochastic resonance processes. However, the mechanisms underlying this general phenomenon remain to be characterized. Here we studied how externally applied noise influences action potential firing in mouse primary sensory neurons of dorsal root ganglia, modelling a basic process in sensory perception. Since noisy mechanical stimuli may cause stochastic fluctuations in receptor potential, we examined the effects of sub-threshold depolarizing current steps with superimposed random fluctuations. We performed whole cell patch clamp recordings in cultured neurons of mouse dorsal root ganglia. Noise was added either before and during the step, or during the depolarizing step only, to focus onto the specific effects of external noise on action potential generation. In both cases, *step + noise* stimuli triggered significantly more action potentials than *steps* alone. The normalized power norm had a clear peak at intermediate noise levels, demonstrating that the phenomenon is driven by stochastic resonance. Spikes evoked in *step + noise* trials occur earlier and show faster rise time as compared to the occasional ones elicited by steps alone. These data suggest that external noise enhances, *via* stochastic resonance, the recruitment of transient voltage-gated Na channels, responsible for action potential firing in response to rapid step-wise depolarizing currents.

## Introduction

In everyday life, sensory stimuli are intrinsically embedded in a noisy environment, as background random signals overlap with the relevant stimulus. To extract information from such a complex input, the nervous system has basically two choices: devise methods to filter noise away or exploit noise to enhance perception.

While neural circuits devoted to sensory filtering have evolved and play crucial roles, at least in mammals (for review, see [1]), the use of noise itself to enhance detection of weak stimuli occurs across the animal realm, from invertebrates [2, 3] to humans [4, 5]. Theoretical and experimental studies on perception of tactile and proprioceptive stimuli show that this phenomenon represents a form of stochastic resonance, as reviewed in [6].

Stochastic resonance is a process whereby superposition of white noise, which contains a wide spectrum of frequencies, onto a periodic signal, in itself too weak to be detected by a sensor, can make it detectable [7]. Such theoretical frame was developed to explain how minimal changes of the eccentricity of the earth's orbit around the sun have driven glaciations cycles in the past million years [8, 9]. Since then, it has found extensive application in neurophysiology [6]. The principal signature of stochastic resonance is that system output shows the frequency of the forcing signal (hence the concept of resonance), provided that noise intensity lies in the range delimited by a bell-shaped curve with a clear maximum. Too small or too large noise amplitudes abolish coupling between stimulus and system response [10]. Also non-periodic stimuli can induce a form of aperiodic stochastic resonance, in which coherence between noisy stimulus and system response can be demonstrated, theoretically and experimentally [11, 12].

Many studies on the importance of stochastic resonance in sensory perception in humans have been carried out applying a noisy stimulus and recording subjects' awareness of the stimulus. In animal studies and in some human studies, including the first on human muscle spindles [4], action potentials evoked by noisy mechanical stimuli in afferent nerves were recorded. However, even in this relatively simplified setting, mechanical noise can exert its effects at multiple levels. For instance noise can alter the mechanical properties of skin or spindle fibres, and thereby the recruitment of sensory nerve endings. Noise could also enhance the function of channels involved in mechano-electric transduction, causing a larger receptor potential, or it could affect action potential initiation at a given receptor potential or a combination of all these factors. Therefore, it remains unclear how each step in the long chain of events that connect mechanical stimulation to perception is affected by stochastic resonance.

Enhanced perception of mechanical stimuli has been obtained in humans also adding electrical noise, either to the same site of mechanical stimulation [13] or more proximally, along the nerve course [14]. This piece of evidence suggests that noise does play a role at the level of action potential generation and conduction, but no experimental proof is available.

Detailed understanding of noise role in tactile and proprioceptive sensitivity is gaining interest as therapeutic interventions based on stochastic resonance are being proposed to treat sensory deficits in a variety of human pathologies (for review, see [15]). Thus, in the present work, we isolated a basic process in the sensory pathway, that is, action potential firing in primary sensory neurons, and explored how it is influenced by external noise under standardized experimental conditions. To obtain stimulation protocols that mimic stochastic fluctuations in receptor potential, likely to be induced by noisy mechanical stimuli, we superimposed electrical noise onto step depolarizing currents.

We performed the experiments in mouse sensory neurons of dorsal root ganglia (DRG) for several reasons. First, the well-established influence of stochastic resonance on tactile and proprioceptive sensitivity (see above) clearly indicates that function of DRG neurons is affected by this phenomenon. Second, these neurons are large, so that intrinsic variability in ion channel function can be neglected [16]. Last but not least, *in vitro* and *in vivo* these neurons do not receive synaptic inputs [17], a feature that contributes to limiting random stimuli to those experimentally applied.

Our data provide proof of principle that external noise increases the excitability of primary sensory neurons by stochastic resonance processes.

## Materials and Methods

### Neuronal Culture Preparation

Procedures using laboratory animals were in accordance with the Italian and European guidelines and were approved by the Italian Ministry of Health in accordance with the guidelines on the ethical use of animals from the European Community Council Directive of 22 September 2010 (2010/63/EU). All efforts were made to minimize the number of animals used and their suffering. Neurons were derived from four months old FVB and C57J mice of both sexes. After deep anaesthesia with halothane, mice were sacrificed by cervical dislocation. The spine was removed and cleaned up from muscle and connective tissue. Twenty to thirty DRGs were isolated from the full length of the spine.

In order to degrade the connective capsule that envelops ganglia they were incubated in papain (20 units/ml) for 20 minutes at 37°C and then in collagenase (5 mg/ml) for 20 minutes at 37°C. Ganglia were then mechanically dissociated, using a glass pipette, and the resultant cells suspension was centrifuged at 300 rpm for 5 minutes. DRG neurons were re-suspended in culture medium consisting of Dulbecco Modified Eagle’s Medium (DMEM) supplemented with 10% Fetal Bovine Serum, 1% antibiotics (penicillin/streptomycin) and Nerve Growth Factor (NGF, 200 ng/ml). The cells were plated in a single drop in the middle of 35 mm Petri dishes coated with laminin (30μg/ml). After 40 minutes, when neurons had adhered to the substrate, 1.5 ml of medium was added to each dish.

### Electrophysiology

Patch-clamp whole-cell recordings were performed 18 to 48 hrs after cell plating using current-clamp mode. Recordings were carried out at room temperature (31-27°C) using an Axopatch 200B amplifier (Molecular Devices, Union City, CA, USA), driven by pCLAMP9.2 (Molecular Devices). Neurons were bathed in standard external solution containing (mM): 140 NaCl, 2.8 KCl, 2 CaCl_2_, 2 MgCl_2_, 10 HEPES/NaOH, 10 glucose, pH 7.3. Patch pipettes made of borosilicate glass (4–7 MΩ tip resistance) contained (mM): 135 Kgluconate, 5 BAPTA, 10 HEPES-KOH, 2 Mg-ATP, 2 MgCl_2_, 5 NaCl, 0.3 Na-GTP pH 7.3. With these solutions, junction potential was +16 mV and was subtracted from all recordings. In all experiments, neurons were continuously superfused using a gravity-driven fast exchanger perfusion system (RSC-200, Bio-Logic, France). Current clamp recordings were routinely performed using the I-clamp NORMAL setting, as indicated by Axopatch 200B manufacturer when the resistance of patch pipettes is less than 10 MΩ. Indeed, direct comparison of recordings obtained using both I-clamp NORMAL and FAST settings in the same neuron shows that spike timing is not affected by the recording modality.

Action potentials were evoked by stimuli lasting 30 or 40 ms, delivered at 1 Hz. To assess the role of noise, groups of 5 current steps or steps plus white noise were applied in alternation. White noise was obtained generating different sequences of pseudo-random numbers with uniform distribution between -1 and 1 (mean = 0, variance = 0.33) using a Mersenne Twister pseudo-random number generator [18]. Noise variance was varied multiplying random numbers by values ranging from 0.125 to 8. Random number sequences were added to steps from 0 to 1 and delivered via a digital-analogue converter (Digidata 1322A, Axon Instruments) to the neurons, with a sampling interval of 0.02 ms, using the "*stimulus file*" option of pClamp. Stimulus amplitude was adjusted in each cell using the "*scale factor*" option, taking care that step amplitude remained just below threshold in repeated tests. Step amplitude ranged from 0.5 nA to 3.5 nA. Noise variance was scaled together with step, so that the ratio between step amplitude and variance remained constant across cells. More than one sequence, each at several variance amplitude, was applied to most cells.

### Data Analysis

**Fig 1:**
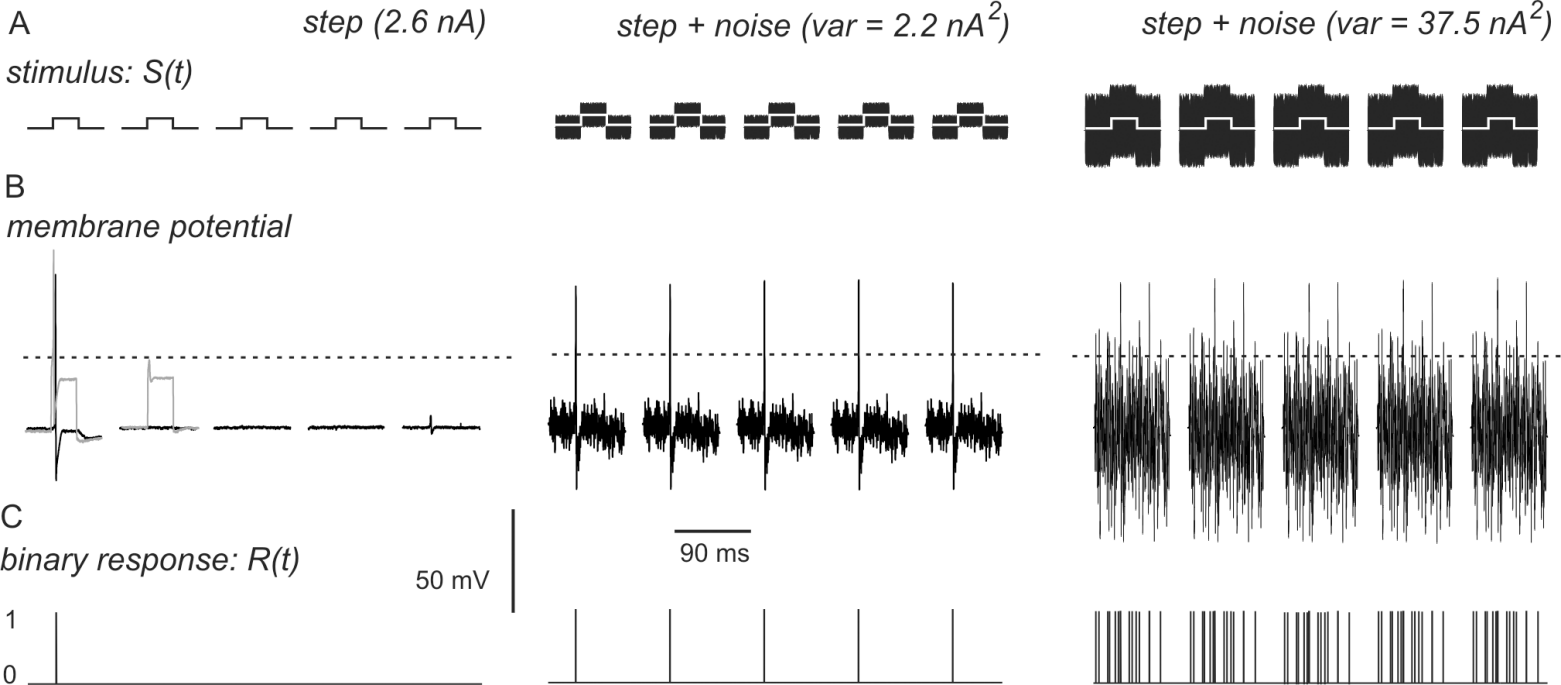
Examples of the functions S(t) and R(t) used to calculate normalized power norm. (A) The stimulus function S(t) consists of sequences of numbers with 0 mean yielding either 5 depolarizing steps (amplitude, 2.6 nA in this case), or steps plus white noise scaled to different variance (*var*). (B) Stimuli evoke passive changes in membrane potential onto which action potentials can be superimposed (grey traces). Subtraction of the passive component yields the net responses (black traces). (C) A binary response function R(t) is calculated form the net response, setting R(t) = 1 when membrane potential exceeds half the peak value of the spike (dashed lines) and R(t) = 0 elsewhere.

A typical measure of coherence between stimulus and response used for aperiodic stochastic resonance is the normalized power norm C_1_ [11, 12], which represents the maximum value of the normalized cross-correlation function and is defined as (overbar indicates average over time):

$$C_{1}=\frac{\bar{S(t)R(t)}}{\sqrt{\bar{S^{2}(t)}}\times \sqrt{{\left[\bar{R(t)-\bar{R(t)}}\right]}^{2}}}$$

where S(t) is the stimulus waveform, built as described above, minus its mean value, as it is assumed to have 0 mean. R(t) is the response of the neurons. Since the biologically relevant signal is action potential firing, we transformed the recorded membrane potential into a binary response function, setting R(t) = 1 when membrane potential exceeded half the peak value of the spike and R(t) = 0 elsewhere (Fig 1).

Action potentials were analysed using Clampfit and NeuroMatic (http://www.neuromatic.thinkrandom.com/) run in Igor Pro (WaveMetrics). Phase plots, representing the first derivative of membrane potential (dV_m_/dt) versus the value of membrane potential at the corresponding time, were built for each action potential [19]. Spike threshold was estimated as the membrane potential value at which dVm/dt increased suddenly and developed with a monotonic rise.

Statistical analysis was performed using Fisher's exact test or Student's *t* test.

## Results

### Stochastic Resonance in Single Neurons

DRGs contain the soma of receptors for all types of somatosensory stimuli, including thermal and painful ones. The effects of stochastic resonance have been established for the perception of tactile and proprioceptive stimuli, which activate receptors with large, myelinated axons of Aα/β group and large cell body [20]. For DRG neurons in culture, the soma diameter of mouse Aα/β-fibre neurons is greater than 30 μm [21].

We therefore studied cells of large diameter and membrane potential negative to -40 mV, which responded to depolarizing currents with overshooting action potentials. Spikes were invariably evoked by stimulations at 1 Hz, whereas some failures occurred when stimulation frequency was increased to 2 Hz (data not shown). Therefore, experiments were performed at 1 Hz. In virtually all neurons tested, only single spikes were evoked when depolarizing steps lasting 20 to 80 ms were applied, both in the presence and in the absence of noise, in line with previous findings in both cultured and acutely isolated DRG neurons [22, 23] Spontaneous firing was detected in only one out of over 100 neurons patched.

**Fig 2:**
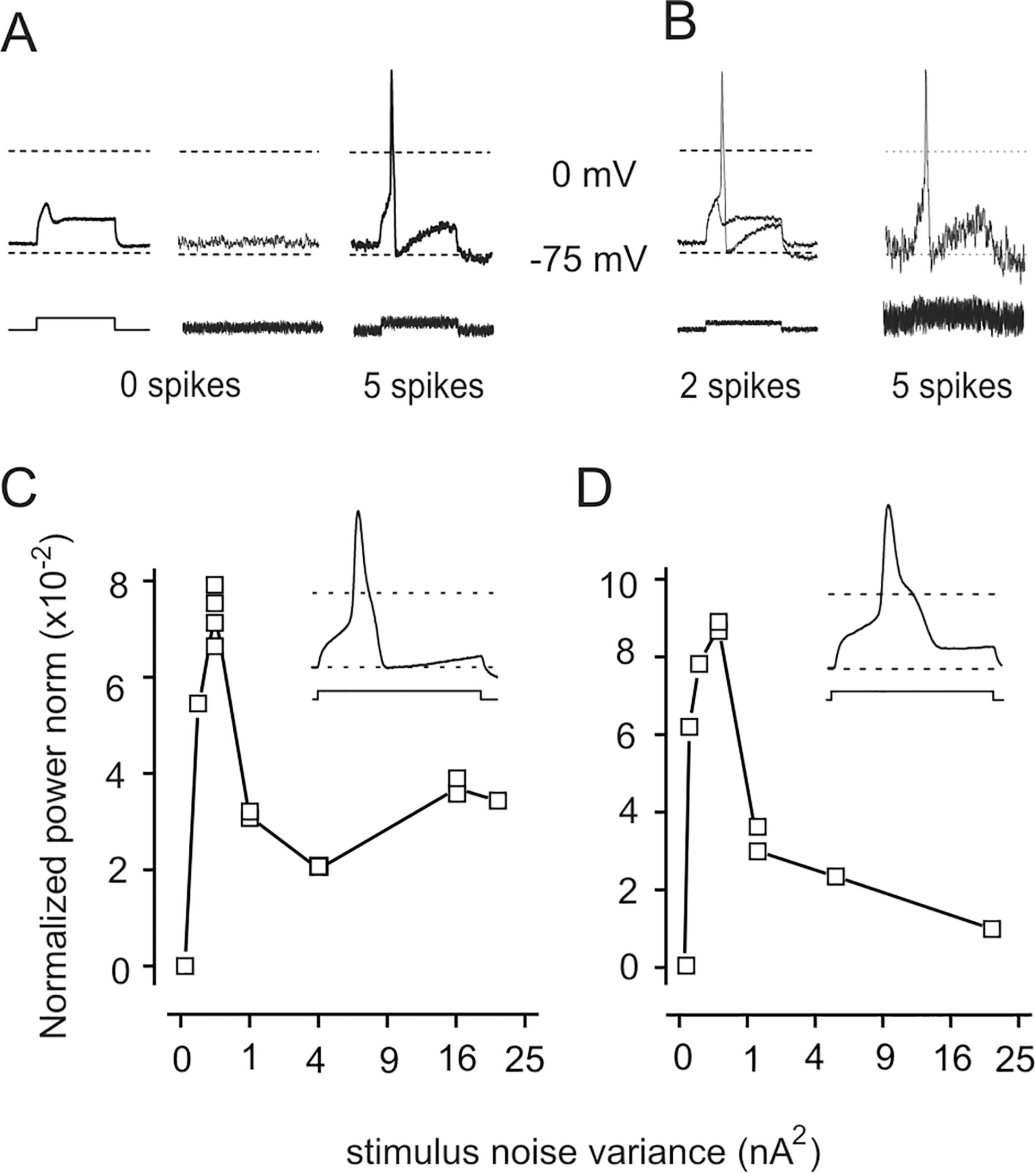
Noise enhances action potential firing in a DRG neuron. (A) *Step* stimuli (left) or noise alone (middle) fail to elicit action potentials (superimposed responses, top traces), while the *step + noise* (right) consistently elicits spikes in all 5 trials in a block. Step duration, 30 ms. (B) In the same neuron, the number of spikes changes if noise amplitude is reduced (left) or increased (right). (C) The normalized power norm C_1_ in the same neuron; each square represents the value obtained in a single block. The clear maximum at intermediate noise variance demonstrates the occurrence of stochastic resonance. *Inset*: The decay phase of action potentials is monotonic in this neuron. (D) In a neuron showing a clear hump in the decay phase of the spike (*inset*), the plot of C_1_ *vs*. noise variance is similar to that presented for the fast neuron in (C)

To investigate the effects of noise, in each neuron we determined the maximal amplitude of depolarising stimuli, which failed to evoke action potentials, so that blocks of 5 depolarizing stimuli consisting of steps only occasionally triggered action potentials (*step* trials; Fig 2A). In the 6 neurons tested, this "subcritical" step amplitude ranged between 0.5 and 3 nA, depending on cell membrane capacitance. Noise alone also failed to evoke spikes in all cells (Fig 2A). Conversely, steps plus noise usually triggered spikes (variance: 0.33 x step amplitude^2^; *step + noise* trials; Fig 2A). In each of the 6 neurons tested, the number of spikes increased significantly in the presence of noise, as determined by Fisher's exact test (Table 1). Overall, spikes were evoked in 27 out of 200 *step* trials, in 147 of 185 *step + noise* trials.

**Table 1:** Responses to depolarizing stimuli in 6 different neurons.

cell	Step		Step + noise		P
	Spikes	Failures	Spikes	Failures	
N1	20	50	37	18	1.3x10^-5^
N2	5	30	40	0	4.0x10^-16^
N3	1	19	20	0	8.0x10^-12^
N4	1	34	20	10	1.7x10^-8^
N5	0	20	8	7	2.7x10^-4^
N6	0	20	17	3	1.3x10^-8^

Number of spikes and failures elicited by *step* or *step + noise* stimuli (noise variance = 0.33 x step amplitude^2^). P value was computed for each neuron using Fisher's exact test.

To test whether the effect of noise is due to stochastic resonance, we measured responses at different values of noise variance. With small noise variance, *steps + noise* evoked spikes only in some trials per group, whilst at larger values spikes were invariably observed (Fig 2B). Thus, the neuronal response consists of 0 to 5 spikes per trial. We quantified the coherence between stimuli and responses by means of the normalized power norm C_1_ introduced for aperiodic stochastic resonance [11]. When noise variance was large enough to trigger spikes in each trial, block–to–block variability for C1 within each neuron was less than 20% (Fig 2C and 2D). In good agreement with stochastic resonance hypothesis, in the 6 neurons tested the normalized power norm C_1_ showed a relatively sharp peak (range: 3×10^−2^ to 11×10^−2^) at small noise variances (range: 0.19 to 0.51 nA^2^) then decreased (Fig 2C). Of note, the range of "resonant" noise variance is narrower than the range of subcritical step amplitude, reinforcing the notion that stochastic resonance occurs. From these experiments, we can conclude that noise promotes action potential firing in response to depolarizing steps in large DRG neurons in culture, through stochastic resonance.

The decay phase of action potentials was fast and monotonic in some neurons (see inset in Fig 2C), whilst it showed a clear hump in others (Fig 2D), which have been proposed to be nociceptors [22]. The hump in the decay correlates with the presence of TTX-insensitive currents [24]. However, plots of C_1_ *vs*. noise variance were quite similar in all neurons tested whatever the shape of the action potential decay (Fig 2D), suggesting that stochastic resonance is a very generalized phenomenon.

### Effect of Noise on Spikes

To focus on the possible influence of noise specifically on action potential triggering, we performed another set of experiments, in which noise was superimposed only on the depolarizing stimulus. This configuration is not strictly conforming to classical stochastic resonance conditions, but eliminates noise-induced effects on neuronal state during inter-stimulus intervals.

**Fig 3:**
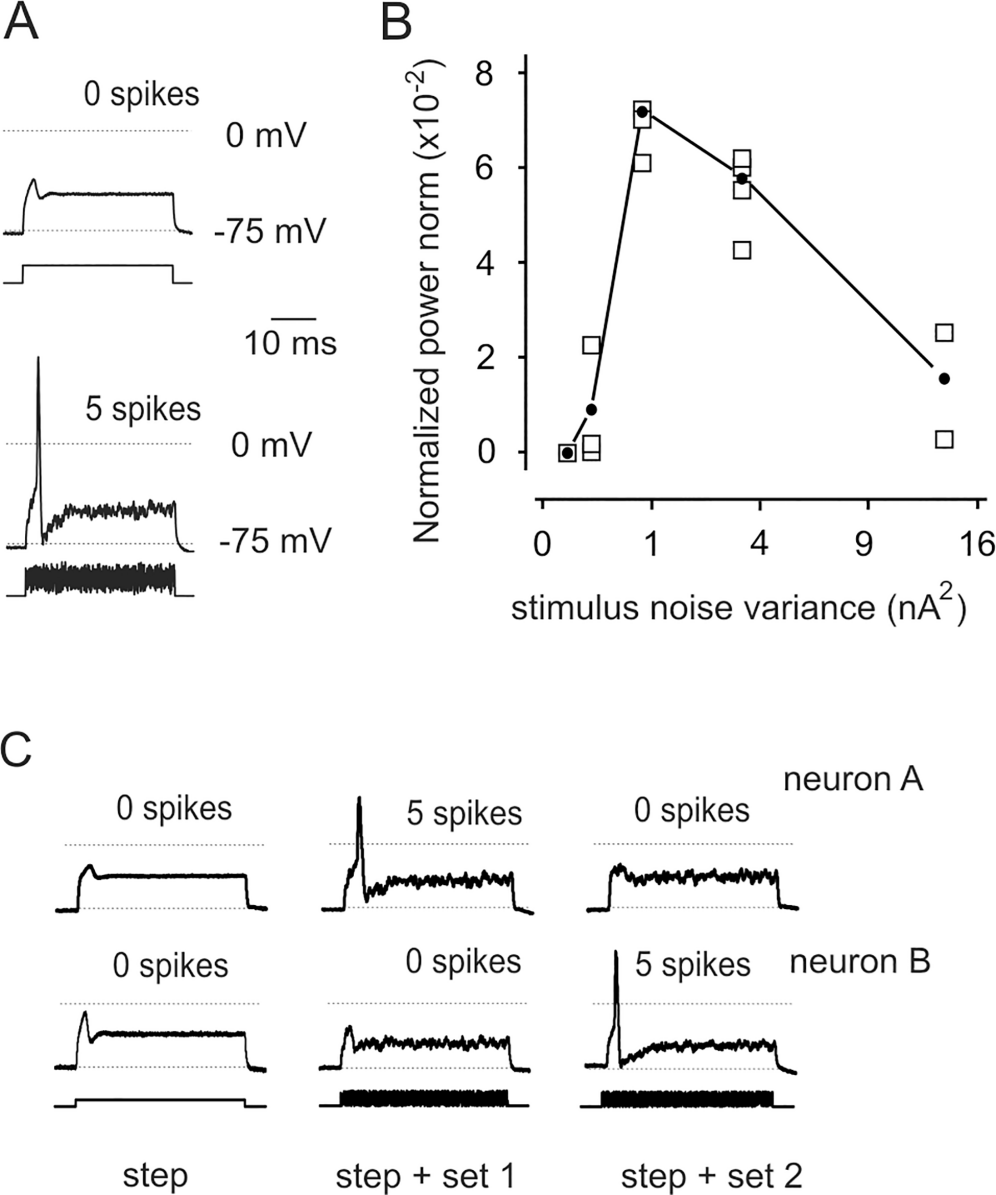
Stochastic resonance occurs when noise is limited to the depolarising phase of the stimulus. (A) Superimposed responses to blocks of 5 stimuli consisting of only *step*, yielding no action potential, or *steps + noise*, yielding 5 spikes. (B) Typical example of normalized power norm C_1_ showing a clear maximum at intermediate noise variance. Squares represent C_1_ values of individual blocks; black dots indicate average values. This curve was obtained from one of the few experiments performed using I-clamp FAST setting. (C) Responses to two sets of random numbers in different neurons, indicating that the effect of noise is not a mere summation onto the step, but rather a real resonance.

Significantly more spikes were evoked by *steps + noise* (variance: 0.33 x step amplitude^2^) than by *steps* alone (Fig 3A) in 33 out of 38 neurons tested, as determined by Fisher's exact test. Overall, in the 38 tested neurons, action potentials were observed in 447 out of 1748 *steps* trials, in 1003 out of 1670 *steps + noise* trials, including all noise realizations applied to each neuron. The normalized power norm showed a distinct maximum (Fig 3B) in the 16 cells tested varying noise variance over a wide range, again indicating that processes attributable to stochastic resonance take place in DRG neurons. C_1_ values had limited block–to–block variability around the peak (Fig 3B). Of note, whilst the presence of noise enhanced spiking, the process appeared to be non-deterministic, as a given noise realization (i.e. sequences of random numbers) might fail to be spike-promoting in few neurons. However, changing the noise realization, the neuron responded to *steps + noise* stimuli (Fig 3C). As above, there was no difference between results obtained in neurons showing "fast" decay or a hump in the decay phase (data not shown).

**Fig 4:**
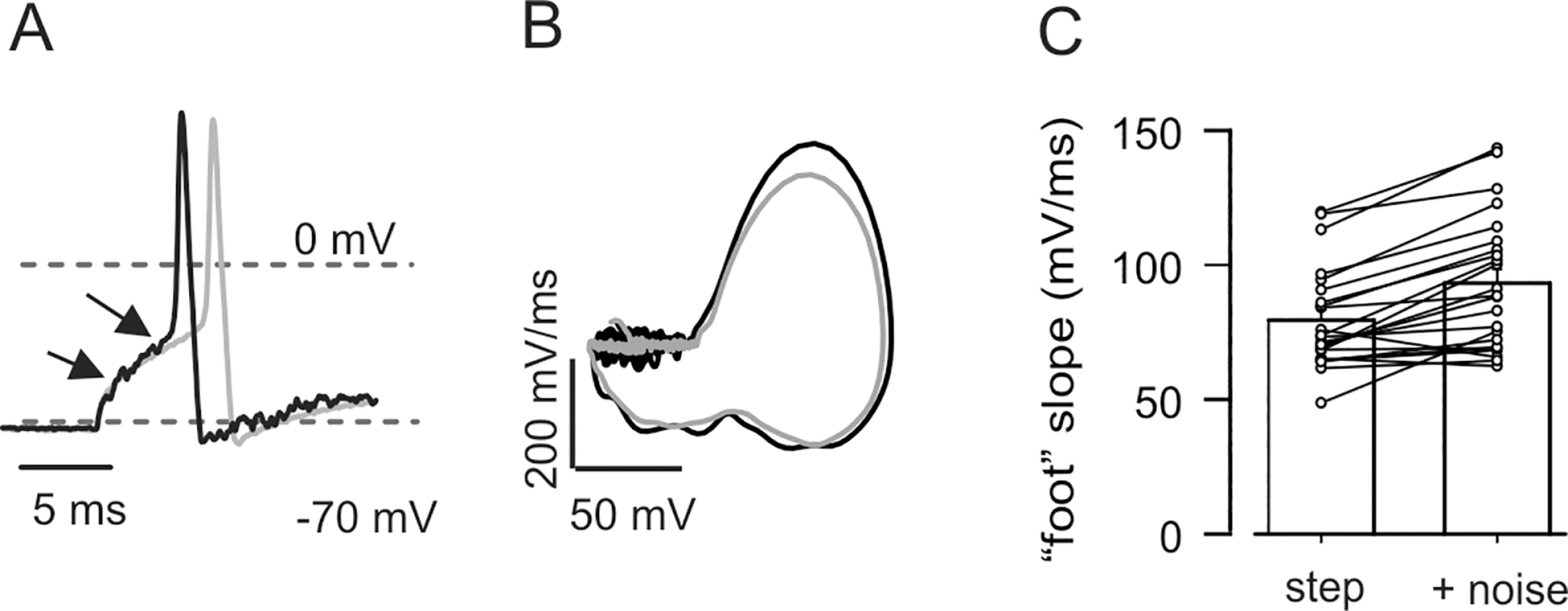
Noise induces acceleration of action potential kinetics in DRG neurons. (A) Superimposed action potentials from a DRG neuron with monotonic decay in response to *step* (grey) and *step + noise* (black) stimuli. Threshold for spike and peak are reached earlier in the presence of noise. (B) Phase plots of the same spikes showing that inflection point (threshold for spike) is almost superimposed in the two curves. (C) With noise, depolarisation to threshold is faster in all neurons tested. Bar graph represents average slope of membrane potential (measured by linear fit) in the region indicated by the arrows in A, with and without noise. Lines represent values in individual neurons (n = 23, P < 0.00005 by paired Student's *t* test).

Comparing the occasional responses to depolarizing steps in control conditions to those evoked by *step + noise* stimuli at equal step amplitude, we can gain insight into which aspects of action potential firing are affected by stochastic resonance. The most evident phenomenon observed in the presence of noise was the shortening of spike latency (Fig 4A); this was detected in all neurons examined (n = 23), independent of the shape of their decay. On average, the spike with the shortest latency was recorded 10.0 ± 0.3 ms after the beginning of the *step* stimuli but already after 9.1 ± 0.3 ms from start of *step + noise* stimuli (P < 0.00002 by paired Student's *t* test). In line with this, the action potential threshold was also reached sooner in the presence of noise, on average by 0.8 ± 0.1 ms (n = 23, P < 0.00002 by paired Student's *t* test). No difference was found in threshold voltage (−27 ± 2 mV for both conditions, P = 0.509), estimated from phase plots (Fig 4B). Conceivably, the observed shortening of the spike latency should depend on the acceleration of the depolarising kinetics. To address this, we measured (by linear fit) the fast, supra-threshold depolarization leading to the peak of the spike (slope of the 20%-80% rise-time) and the slow component of the sub-threshold membrane depolarization in response to injected current (the spike ‘foot’, leading to the threshold). As predicted, both these phases were accelerated in the presence of noise: the supra-threshold rate of rise increased by 6% (*step*: 247 ± 15 mV/ms, *step + noise*: 262 ± 15 mV/ms; n = 23, P < 0.0003 by paired Student's *t* test); the sub-threshold component increased by 17% (*step*: 80 ± 4 mV/ms, *step + noise*: 93 ± 5 mV/ms; n = 23, P = 0.00001 by paired Student's *t* test. Fig 4C). These kinetic effects were independent of the shape of action potential decay (that is, fast or with hump; unpaired Student's *t* test). The decay phase of fast-falling spikes was only modestly accelerated, by 2 mV/ms (n = 17, P = 0.047 by paired Student's *t* test).

## Discussion

The role of stochastic resonance in enhancing sensitivity to mechanical stimuli is well documented, but the mechanistic aspects of this phenomenon remain poorly defined. We focused our attention onto a model of a single step of sensory perception: action potential firing in primary sensory neurons. Reasoning that noisy mechanical stimuli may cause stochastic fluctuations in receptor potential, we examined the effects of sub-threshold depolarizing current steps with superimposed electrical white noise. We show that these *step + noise* stimuli trigger action potentials in primary sensory neurons when *steps* alone fail, and that the phenomenon is driven by stochastic resonance, as demonstrated by the dependence on noise variance. By superimposing noise only on the depolarizing current, we could focus onto the specific effects of external noise on action potential generation. Significantly more action potentials are triggered by *step + noise* stimuli than by steps alone. Concomitantly, spikes evoked in *step + noise* trials occur earlier and show a slightly faster rise time as compared to the occasional ones elicited by steps alone. These data suggest that external noise enhances, *via* stochastic resonance, the recruitment of transient voltage-gated Na channels, responsible for action potential firing in response to rapid step-wise depolarizing currents [25]. The small acceleration of the decay phase could reflect a corresponding anticipation of Na channels inactivation. A contribution to the accelerated onset of spikes could derive from a reduction of K^+^ conductance [25]. Such a process however would lead to a slowed repolarisation, at odds with our data.

The results provide proof-of-principle that fluctuations in receptor generator potential, possibly induced by noise in mechanical stimulation, can facilitate spike generation in primary sensory neurons. Such an event would be prerequisite for the enhanced detection of mechanical stimuli in the presence of noise, observed in animals and humans.

An apparent drawback of this work is that we studied–*in vitro*–somatic action potentials, whilst sensory transduction takes place in nerve endings and action potentials are generated in the axon, at the first node of Ranvier, and propagated towards the soma and the spinal cord (see for instance [26]). We can imagine only three possible *a priori* arguments against the hypothesis that our results extend to the physiological case: that different channels sustain action potentials in the soma and in the node of Ranvier; that similar channels are present in extremely different amounts; that noise amplitude is too large to be realistic. Several isoforms of Na channels have been identified in DRG neurons, yielding currents with different functional properties, including sensitivity to tetrodotoxin. Tetorodotoxin-insensitive currents appear to be more represented in nociceptive neurons and correlate with the presence of a hump in the spike repolarizing phase [24]. Since we found no difference in the effects of noise in neurons with different action potential shapes (and presumably, different function), we can assume that the molecular identity of Na channels is irrelevant to stochastic resonance processes. In other words, noise effect on the function of sensory neurons is a very general phenomenon. Coming to the second point, theoretical calculations indicate that groups of less than about 3000 channels are dominated by intrinsic noise and relatively insensitive to external noise, so that stochastic resonance phenomena are restricted to large channel assemblies [16]. Voltage-gated Na channels are extremely numerous at nodes of Ranvier of mammalian nerves, with estimates as high as 700000 per node [27]. In the soma of cultured DRG neurons, total number of channels arguably is in the same order of magnitude. Our cursory analysis of voltage gated Na^+^ currents in few neurons revealed that current amplitude was too large for adequate voltage clamp, consistently being larger than 50 nA at—20 mV. Considering that unitary conductance of voltage-gated Na channels has been reported to range between 10 and 15 pS in cultured DRG cells [28], more than 50000 non-inactivated channels must be present to yield the recorded currents. As for the amplitude of noise, it must be noted that in the experiments here reported, noise amplitude was relative to that of the step. In other words, when small stimuli were sufficient to elicit spikes, noise amplitude was correspondingly small. Thus, there is no reason to believe that the described effect cannot occur under physiological conditions. This extrapolation is further supported by the widespread influence of stochastic resonance processes in sensory transduction, occurring, for different sensory modalities, from invertebrates to humans, as outlined in the Introduction.

To our knowledge, this is the first experimental demonstration that external white noise can boost, through stochastic resonance, the recruitment of ion channels directly responsible for action potential generation in neurons. To date, stochastic resonance with externally applied white noise has been demonstrated to favour the opening of channels formed by the antibiotic alamethicin in a lipid bilayer [29]. In biological systems, evidence for stochastic resonance has been provided for membrane excitability as a whole (see for instance [30, 31]) or through action on constitutively active currents. For instance, external white noise has been shown to enhance sub-threshold spontaneous membrane potential oscillations in injured DRG neurons [32], which depend on the persistent Na^+^ current, spontaneously active in a subset of injured DRG neurons [33]. It has also been demonstrated that the inherently stochastic nature of the persistent Na^+^ current (termed "internal noise") strongly influences the activity of stellate neurons of the entorhinal cortex *via* stochastic resonance [34, 35]. However, spontaneous firing is very rare in DRG neurons [36], so we show that external noise influences activation of channels by external stimuli.

In summary, we provide evidence that external noise boosts action potential firing in primary sensory neurons, therefore establishing at least one mechanism whereby noise can enhance detection of weak mechanical stimuli.
